# China: leapfrogging to become a leader in global health?

**DOI:** 10.7189/jogh.09.010312

**Published:** 2019-06

**Authors:** Titiporn Tuangratananon, Kun Tang, Rapeepong Suphanchaimat, Viroj Tangcharoensathien, Suwit Wibulpolprasert

**Affiliations:** 1 International Health Policy Program, Ministry of Public Health, Nonthaburi, Thailand; 2 Department of Global Health, Peking University, Beijing, China; 3 Bureau of Epidemiology, Department of Disease Control, Ministry of Public Health, Nonthaburi, Thailand

**Figure:**
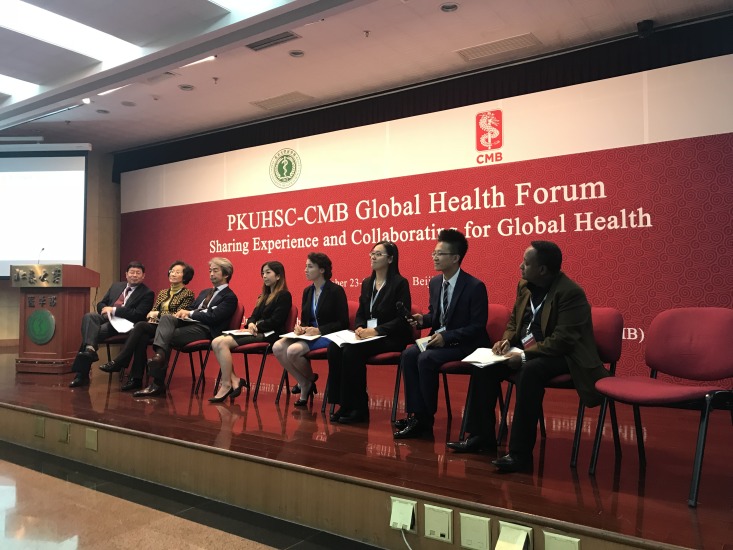
Photo: Global health experts meeting in Beijing, 2017 (from the collection of Titiporn Tuangratananon, used with permission).

China, the emerging economic giant in the world, is now playing an active role in the arena of global health. It is active in bilateral collaboration, South-South collaboration and the Belt Road Initiative, and has dispatched medical teams, built infrastructure and provided assistance with health technology overseas. Despite its bilateral health initiatives, China has invested little in established multilateralism mechanisms. Although several university global health institutes have been established, China’s participation on the global health stage, such as at the World Health Assembly, has been limited. Global health leaders require capabilities beyond financial resources, and human resources inevitably play a critical role. Limited global health professional capacity in China is a challenge. Alongside global health strengthening, in-country health systems development is also essential. China, a country of 1.4 billion people, has invested in universal coverage and health security with some challenges still remaining; however, success would be a valuable lesson to the world of global health. Gigantic China with successful universal health coverage could shift the existing global paradigm. While challenges need to be overcome, with collective effort and strong political commitment China is positioned if it wishes to become the next leader in global health. China’s impact will hugely benefit not only the Chinese people but all peoples around the world.

## CHINA: EMERGING ON THE GLOBAL HEALTH STAGE

Global health, a recently new term replacing ‘international health’, is not difficult to understand but it can be interpreted and implemented differently across countries. Global health emphasizes transnational health issues, determinants and solutions. It involved many disciplines within and beyond the health sciences and promotes interdisciplinary collaboration. It is also recognized as a synthesis of population-based prevention combined with individual-level clinical care [1].

Countries take up different positions in the global health arena, especially emerging powers, such as China. Undeniably, evidence shows that China has experienced impressive growth in all dimensions from economics, science and technology, social advancements to geo-political power. In the past three decades, China’s Gross Domestic Product (GDP) growth rate has remarkably increased by 10 percent every two years [2]. China is also taking the lead in energy conservation technology to tackle climate change and strengthening international collaboration through investment and economic support [3]. The country is currently the second largest economy in the world, by nominal dollars and the largest economy by purchasing power parity [4]. The question now is how will the China with its giant economy influence global health?

In global health, China is considered a muscular country, that can move the agenda forward. China has played active roles in development, including health, in many foreign countries for decades. It has been one of the key pioneer investors in Africa and is taking the lead elsewhere too. One key example is the “Belt and Road Initiative” which aims to boost trade and economic growth across Asia and beyond.

Political commitment plays a very vital role. Recently, President Xi Jinping stated in the 19th National Congress of the Communist Party of China that China will be, “Moving closer to the center stage, and making a greater contribution to mankind” [5]. These trends are returning China to the ‘global leader’ it once was before the 19th century. It is obvious that China is an emerging power in the Post-American or Pax Americana era. What is China’s global vision of health progress for humanity.

## WHAT HAS CHINA CONTRIBUTED TO GLOBAL HEALTH?

In health, China has its own unique collaborative approach with other developing countries. In the past six decades, China’s global health policy has focused on bilateral collaboration such as dispatching medical teams to African countries. In 1963, Algeria was the first African country to which the Chinese government dispatched their medical personnel. Motives behind this collaboration were analyzed in a number of publications and China’s foreign policy in the Cold War era might have been the most important attribute at that time. The export of socialist experience and political directions against Western sanction are also key underlying motives. The State council of China pointed out that China was also be able to reap economic benefits as a by-product from this aid [6,7].

China’s medical assistance to Africa mainly focused on infrastructure investment, provision of medical supplies, dispatching medical teams and financial support. Although China has invested in bilateral collaboration for 60 years, the annual budget for a single African country can be as modest as US$ 1.8 million [8]. The total foreign aid budget in 2009 was US$ 37.52 billion; of this, only 6% was for health care grants and 3% was for health loans. This reflects a lower priority for health care support compared to other sectors [8]. As well as bilateral collaboration, China is embarked on new multilateralism mechanisms. The total budget between 2010 and 2013 was US$ 1.644 billion. This continues to grow following the ‘South-South Collaboration Fund’ strategies [9], and Fund recipients extend beyond Africa to fifty countries worldwide. Nevertheless, these forms of health aid are still similar to bilateral collaborations [10].

A similar pattern was observed in Brazil, Russia, India, China and South Africa (BRICS) where there appeared to be modest amounts of financial aid. The countries positioned themselves as ‘partners’ instead of “donors”. South-South collaboration illustrates this partnership approach. Technology transfer, technical assistance and empowering partnerships are the key messages. The difference between BRICS and China is that BRICS show relatively less interest in offering financial investments to key international development partners that include the Global Fund, the GAVI Alliance, or the World Health Organization (WHO) [11].

Chinese investment in multilateral mechanisms, like WHO and Global fund, is however small relative to its investment in bilateral collaborations. In the 2016-2017 WHO biennial budget, China contributed US$ 60.7 million for an assessed contribution, and made a voluntary contribution of approximately US$ 8.2 million (one seventh of its assessed contribution). The proportion of voluntary contributions to assessed contribution given to WHO by a number of high-income countries were much larger than that made by China (for example, Canada’s proportion was 1.5:1, the US’s was 2:1, and the UK’s was 3:1) [12]. WHO, as the global health governance body, is now dependent more on funding by donors than by member states. About 80% of the WHO budget is financed by donor contributions, and is mostly ear-marked for certain activities [13].

Interestingly, China has committed a new financial contribution to WHO and is strengthening the WHO-China cooperation through the Belt and Road Initiative, announced during the high-level meeting with Dr Tedros Adhanom Ghebreyesus, Director General of WHO, in August 2017. Results from the discussion among domestic and international experts on the panel revealed that China may prioritize “its own” innovative collaboration - for instance South-South collaboration and Belt and road initiative - over the so-called conventional methods’ routinely exercised by WHO, GAVI and Global Fund.

## STEPS TO BE A GLOBAL HEALTH LEADER?

China's overall global health capacity remains limited and it needs to overcome many challenges in order to become a leader in global health. Although China has accelerated the growth of its global health capacity by establishing 19 global health institutes, most of them mainly focus just on general academic activities. A comprehensive assessment and strengthening of their curricular contents, faculty capacities, and training outcomes will definitely be helpful in boosting domestic capacities in global health.

Playing an active role in the World Health Assembly (WHA) mirrors a country’s global health capacity. Although in the past, China sent many young leaders and graduates to attend the WHA (for example it sent 82 delegates to the 70th WHA, which was top among member states). These attendees played mainly technical advisory roles, and fewer active roles like delivering interventions and negotiations on draft resolutions. (China delegates delivered only 29 interventions in the 70th WHA.) Their practice has not been fully institutionalized. In contrast, Thailand, a much smaller country, sent 55 delegates, delivered 59 interventions and played active roles in negotiating draft resolutions. China has always played low-key roles in terms of the number of interventions, tabled resolutions and proposed agendas, but needs to overcome this to fulfil the role of global health leader and institutionalise its practice of influence. Noteworthy is that initiatives by a UN “big 5” country can be expected to be received differently than a medium-sized middle-income country like Thailand.

China is also under-represented in the structure of WHO staff. Although the former DG of WHO, Margaret Chan is from China, she is actually from Hong Kong (Special Administrative Region). Such limitations in human resources and financial support could constrain China from being a global health leader. This however, does not mean that China cannot overcome these challenges and rise to become a leader in global health. The country is now devising many strategies to strengthen its global health capacity. China grants scholarships to students to gain experience abroad and to understand foreign contexts. In addition, it has also carried out many domestic global health workshops. However, it would benefit much more if these global health workshops were based on the country-level experiences of global health, rather than on youngsters trained in western universities or policies dependent upon western advice.

China might be interested in the INNE model (Individual-Node-Network-Environment) which Thailand has used to build its own global health capacity. Based on this model, the Thai Government has set up a global health diplomacy workshop which trains and send novices to the WHA every year [14]. The model is institutionalized within the Global Health Division and the International Health Policy Program in the Ministry of Public Health and connects closely with the Ministry of Foreign Affairs and experts from other countries. Accordingly, the INNE model acts as collective long-term capacity building for the whole country, not just a few global health leaders. The beginnings of this model was tested by sending young scholars by Peking University working in cooperation with the China Commission on Health.

To be more concrete, China might consider focusing on a few select global health topics and enable the world to recognize China as a champion for those issues. One of these is UHC, a vital issue and one where China can make important contributions and changes to the world. Currently, China’s UHC has covered almost 97% of its 1.3 billion population, which is a genuine “global break-through”.

Strong political commitment and leadership have contributed to a continuous development of three main public insurance schemes. Of course, the path towards UHC of China is not straightforward. Several challenges are yet to be addressed, including health inequity between urban and rural populations, poor quality health care services in some areas, and skyrocketing health care expenditure with inadequate cost control. Having recognized these challenges, China is now heading forward a reform in its strategic purchasing, and will hopefully make this a successful show case to the world. Furthermore, successes in strengthening capacity on the control of emerging infectious diseases are quite significant and a Chinese team has already demonstrated its capacity to support the control of Ebola outbreaks in West Africa.

## HEALTHY CHINA 2030: A VISION THAT MAY CHANGE THE WORLD

The Healthy China 2030 vision consists of five major targets to improve the level of health, control major risk factors, increase health service capacity, expand health industry scale, and perfect the health service system. A tremendous investment of 16 trillion yuan (US$ 2.4 trillion) in the services industry confirms a strong political commitment to move the agenda. These targets can significantly contribute to ‘better health’ globally. Access to low-cost-effective medicine is one of many successful examples that China has contributed, and will further contribute, to the world [15]. China has immense capacity to develop essential medicines and previous key examples are Artemisinin and Japanese Encephalitis vaccines. Artemisinin is a life-saving anti-malarial drug discovered and developed by China, without any patent. As a consequence, it is available to many countries at a low and affordable price. China also provides Artemisinin and other anti-malarial drugs to more than 30 malarial centers with the total cost around US$ 30 million per year.

## CONCLUSION

Undoubtedly, China is one of the largest global economic leaders and has the potential to becoming a global health leader, going beyond simply financial contributions. A more comprehensive collaborative strategy to rapidly build China’s global health capacity is the missing piece of a more powerful China engagement. Bilateral collaboration between China and African countries has contributed to significant health benefits, however, more strategic planning and investment in multilateralism, which would benefit the world, needs more attention. Despite remaining challenges, China has shown great success in UHC, and by moving towards Healthy China 2030, it can surely be a role model for, and a partner of, many developing countries in order to drive important global health agendas. With such success, the world will not be able to resist naming China as a ‘global health leader’ in the near future.
